# Association of midlife stroke risk with structural brain integrity and memory performance at older ages: a longitudinal cohort study

**DOI:** 10.1093/braincomms/fcaa026

**Published:** 2020-03-07

**Authors:** Enikő Zsoldos, Abda Mahmood, Nicola Filippini, Sana Suri, Verena Heise, Ludovica Griffanti, Clare E Mackay, Archana Singh-Manoux, Mika Kivimäki, Klaus P Ebmeier

**Affiliations:** f1 Department of Psychiatry, University of Oxford, Warneford Hospital, Oxford OX3 7JX, UK; f2 Wellcome Centre for Integrative Neuroimaging, Oxford Centre for Functional MRI of the Brain, Nuffield Department of Clinical Neurosciences, University of Oxford, John Radcliffe Hospital, Oxford OX3 9DU, UK; f3 Wellcome Centre for Integrative Neuroimaging, Oxford Centre for Human Brain Activity, University of Oxford, Warneford Hospital, Oxford OX3 7JX, UK; f4 Nuffield Department of Population Health, University of Oxford, Big Data Institute, Oxford OX3 7LF, UK; f5 Department of Epidemiology and Public Health, University College London, London WC1E 7HB, UK; f6 INSERM, U1153, Epidemiology of Ageing and Neurodegenerative diseases, Université de Paris, Paris, France

**Keywords:** Framingham stroke risk, cardiovascular health, brain health, structural brain integrity, cognition

## Abstract

Cardiovascular health in midlife is an established risk factor for cognitive function later in life. Knowing mechanisms of this association may allow preventative steps to be taken to preserve brain health and cognitive performance in older age. In this study, we investigated the association of the Framingham stroke-risk score, a validated multifactorial predictor of 10-year risk of stroke, with brain measures and cognitive performance in stroke-free individuals. We used a large (*N* = 800) longitudinal cohort of community-dwelling adults of the Whitehall II imaging sub-study with no obvious structural brain abnormalities, who had Framingham stroke risk measured five times between 1991 and 2013 and MRI measures of structural integrity, and cognitive function performed between 2012 and 2016 [baseline mean age 47.9 (5.2) years, range 39.7–62.7 years; MRI mean age 69.81 (5.2) years, range 60.3–84.6 years; 80.6% men]. Unadjusted linear associations were assessed between the Framingham stroke-risk score in each wave and voxelwise grey matter density, fractional anisotropy and mean diffusivity at follow-up. These analyses were repeated including socio-demographic confounders as well as stroke risk in previous waves to examine the effect of residual risk acquired between waves. Finally, we used structural equation modelling to assess whether stroke risk negatively affects cognitive performance via specific brain measures. Higher unadjusted stroke risk measured at each of the five waves over 20 years prior to the MRI scan was associated with lower voxelwise grey and white matter measures. After adjusting for socio-demographic variables, higher stroke risk from 1991 to 2009 was associated with lower grey matter volume in the medial temporal lobe. Higher stroke risk from 1997 to 2013 was associated with lower fractional anisotropy along the corpus callosum. In addition, higher stroke risk from 2012 to 2013, sequentially adjusted for risk measured in 1991–94, 1997–98 and 2002–04 (i.e. ‘residual risks’ acquired from the time of these examinations onwards), was associated with widespread lower fractional anisotropy, and lower grey matter volume in sub-neocortical structures. Structural equation modelling suggested that such reductions in brain integrity were associated with cognitive impairment. These findings highlight the importance of considering cerebrovascular health in midlife as important for brain integrity and cognitive function later in life (ClinicalTrials.gov Identifier: NCT03335696).

## Teaser

Higher stroke-risk score over 20 years before the magnetic resonance imaging scan was associated with whole-brain grey matter volume and white matter microstructure in community-dwelling older adults. Structural equation models suggest that brain integrity was associated with cognitive function. Results highlight the need for targeting modifiable risk factors in midlife.

## Graphical Abstract

**Graphical Abstract:**
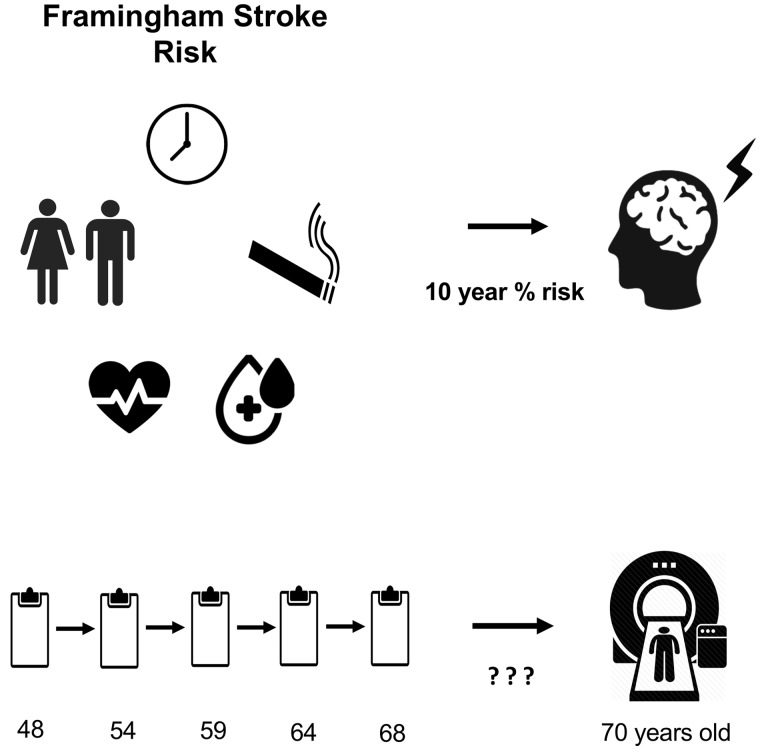

## Introduction

The Framingham stroke-risk score (FSRS) is a multifactorial predictor of 10-year risk of stroke (D’Agostino *et al.*, 1994). It includes cardiovascular, metabolic and health measures and is routinely used in clinical practice to predict the risk of stroke and the need for prophylaxis. Higher FSRS is also linked with lower grey and white matter (WM) integrity in older age (Pase *et al.*, 2018; Zsoldos *et al.*, 2018), lower fractional anisotropy (FA) in late-onset depression (Allan *et al.*, 2012), cerebral small-vessel disease progression and cognitive decline in middle-aged hypertensive patients (Uiterwijk *et al.*, 2018), cognitive decline in the over 50s (Dregan *et al.*, 2013) and poor cognition in older individuals even in the absence of Alzheimer’s biomarkers or neuropathology (Hohman *et al.*, 2015). While individual vascular risk factors of stroke, such as hypertension, atrial fibrillation, diabetes, smoking and obesity in midlife, have been linked with an increased rate of progression of vascular brain injury (Allan *et al.*, 2015), various brain measures (Debette *et al.*, 2011) and poor cognitive performance (Dregan *et al.*, 2013; Kaffashian *et al.*, 2013*b*; Nishtala *et al.*, 2018; Moran *et al.*, 2019), the combined effects of particular vascular risk factors may expedite the process of cognitive decline (Dregan *et al.*, 2013; Kaffashian *et al.*, 2013*a*; Cox *et al.*, 2019). For example, findings from Dregan *et al.* imply that, while cigarette smoking is categorically associated with global cognition, memory and executive function, age and duration of high systolic blood pressure levels have a cumulative, detrimental long-term effect on cognition over time. It has been recommended that interventions to limit cognitive decline should target multiple vascular risk factors rather than manage individual risk (Dregan *et al.*, 2013). It is therefore imperative to understand if cerebrovascular health in midlife is important for brain integrity and cognitive function later in life.

The aim of this study was to assess whether Framingham stroke risk over a 20-year period before the magnetic resonance imaging (MRI) scan was associated with structural brain integrity measures, even after accounting for the effects of confounding variables, such as age (Uiterwijk *et al.*, 2018). We investigated which grey and WM structures were affected by new stroke risk acquired between the study waves. We also examined whether FSRS-predicted cognitive performance was mediated by structural brain measures. Our hypothesis was that higher FSRS will be associated with lower cognitive performance and this association is mediated by lower structural brain measures.

## Materials and methods

### Participant characteristics

A total of 800 Whitehall II participants were randomly recruited from the 2012–13 wave of the study to take part in the Whitehall II imaging sub-study between April 2012 and December 2016 where they underwent multi-modal MRI scanning and neuropsychological testing (Filippini *et al.*, 2014). At study inception (1985–88), the Whitehall II study included 10 308 British civil service workers aged 35–55 years (born between 1932 and 1955), of whom 6895 were men. Follow-up health examinations were conducted over the following 30 years, approximately every 5 years. The present analysis uses data from 1991 to 1994, 1997 to 1999, 2002 to 2004, 2007 to 2009 and 2012 to 2013. A total of 74% of participants who took part at the 2012–13 wave had reached or passed the statutory retirement age of 65 years. Ethical approval was obtained from the University of Oxford Medical Sciences Interdivisional Research Ethics Committee (Reference: MS IDREC-C1-2011-71) and the University College London Committee on the Ethics of Human Research (Reference: 85/0938). All participants provided informed written consent.

### Inclusion/exclusion criteria

Detailed information is provided in the Supplementary Material. In brief, participants were excluded from analysis if they did not have an MRI scan, had obvious structural abnormalities, for example suggesting a stroke, or poor image quality that pre-processing and artefact correction could not fix, or had a missing FSRS at any wave.

### MRI acquisition and analysis

T_1_-weighted, fluid-attenuated inversion recovery (a modified T_2_-weighted sequence) and diffusion-weighted MRI images were acquired at the Oxford Centre for Functional MRI of the Brain, Wellcome Centre for Integrative Neuroimaging. The first 550 participants were scanned on a 3-T Siemens MAGNETOM Verio (Erlangen, Germany) scanner with a 32-channel receive head coil (between April 2012 and December 2014), and due to a scanner upgrade, the last 250 participants were scanned on a 3-T Siemens MAGNETOM Prisma scanner with 64-channel receive head–neck coil (between July 2015 and December 2016) (for sequence parameters, see Supplementary Table 1). All images were processed and analysed using FMRIB Software Library v.6.0 tools (Smith *et al.*, 2004) or FreeSurfer version 5.3. Full technical details are given in the Supplementary Material.

In brief, cortical atrophy was estimated by scaling the grey matter (GM) values for the total intracranial volume (GM + WM + CSF) resulting in percentage total GM volume. Hippocampal volume was also estimated. WM lesions appear brighter (hyperintense) on T_2_-weighted images and are attributed to degenerative changes in small, deep penetrating arteries (Wardlaw *et al.*, 2013), cardiovascular risk factors and age (de Leeuw *et al.*, 2001; Li *et al.*, 2013). WM hyperintensities (WMHs) were automatically segmented on fluid -attenuated inversion recovery images (Griffanti *et al.*, 2016). Voxelwise analysis of GM density was performed using FMRIB Software Library-VBM (Douaud *et al.*, 2007), an optimized voxel-based morphometry protocol (Good *et al.*, 2001). Diffusion tensor imaging quantifies the directionality and rate of diffusion of water molecules within different tissues and allows inferences about the structural integrity of WM tracts. When movement is anisotropic, such as in healthy myelinated fibres, diffusion is restricted perpendicular to the longitudinal axis of the fibre. We carried out voxelwise analysis of diffusion tensor data [FA and mean diffusivity (MD)] with tract-based spatial statistics.

### Framingham stroke-risk score

The FSRS is a stroke-risk appraisal function that empirically relates cardiovascular risk factors to the probability of a stroke within 10 years (D’Agostino *et al.*, 1994). The probability of stroke depends on an individual’s presence and level of risk factors and is expressed as a percentage score. Risk factors include cardiovascular health (systolic blood pressure, prior cardiovascular disease, atrial fibrillation, left ventricular hypertrophy and antihypertensive medication), diabetes mellitus, smoking habits, sex and age. The percentage risk score was computed using beta coefficients based on the Cox proportional hazards regression model in the Framingham study at each data wave (further details in the Supplementary Material).

### Assessment of cognition and premorbid functioning

The Hopkins Verbal Learning Test-Revised (Brandt, 1991) and Test of Premorbid Functioning (Wechsler, 2011) were administered by a trained psychology graduate on the day of the MRI scan, prior to the scan (details in the Supplementary Material).

### Assessment of confounding variables

Age (linear and quadratic term) at the time of scan, sex, ethnicity, education, employment grade and scanner type were used as confounding variables. Ethnicity was limited to white versus non-white. Education years were calculated as the difference between the age at which the participant commenced primary school and the age at which they first left full-time education. Socio-economic status was classified according to the occupation grade between 1985 and 1988: senior managers and administrators (highest grade), professionals and executives (middle grade) and clerical and support staff (lowest grade). Scanner model was defined as Siemens 3-T Verio versus Prisma.

### Statistical analysis

Voxelwise general linear models were generated for the analysis of GM density, FA and MD data using ‘Randomise’ (Winkler *et al.*, 2014), a permutation-based non-parametric statistical programme, running 5000 permutations and correcting for multiple comparisons across space with *P* < 0.05, using threshold-free cluster enhancement (Smith and Nichols, 2009). We used the Harvard–Oxford cortical and sub-cortical structural atlases for VBM and the John Hopkins University diffusion tensor imaging-based WM atlases for tract-based spatial statistics.

Model I: linear associations were assessed between FSRS in each of the five waves and voxelwise GM, and diffusion tensor imaging data, including MRI scanner type as a confounding variable.

Model II: Model I analyses were repeated including all confounding variables.

Model III: results for associations between FSRS at 2012–13 and voxelwise GM, FA and MD were repeated using scanner type and FSRS at 1991–93, 1997–99, 2002–03 or 2007–09 as confounders, to remove between-subject variability including age and sex and examine the effect of residual risk acquired over a 5-, 10-, 15- and 20-year period before the scan.

Model IV: scanner type, percentage GM and WMH volumes were entered as confounding variables to test whether the relationship between FSRS between 2012 and 2013 and lower FA was mediated by percentage GM (i.e. an estimate of cortical atrophy and presumed Wallerian degeneration) or WMH volume (i.e. presumed vascular lesion, as opposed to WM rarefication originating from GM loss).

### Structural equation modelling

IBM SPSS Amos version 25 structural equation modelling software was employed to build additional models to test the hypothesis that stroke risk negatively affects cognitive performance via specific brain measures. Models were optimized by backwards removal of non-significant effects until all effects were significant. With the removal of each variable, the measures of goodness of fit improved. All available data in the covariance matrix were used.

### Data availability

The study follows Medical Research Council data-sharing policies (https://www.mrc.ac.uk/research/policies-and-guidance-for-researchers/data-sharing/, 20 March 2020, date last accessed). Data will be accessible from the authors after 2019.

## Results

### Descriptive statistics

#### Participant exclusion/inclusion

VBM analysis was based on a final available sample of *N* = 566, tract-based spatial statistics on *N* = 548 and structural equation modelling on *N* = 775 (for details of exclusions, see Supplementary Material).

#### Socio-demographic variables

The mean participant age at the time of scan and during the previous study waves is listed in Table 1; 80.6% of participants were male, with on average 14 years of education, reflecting the demographics of the British Civil Service at recruitment to the Whitehall II study in 1985 (Table 2). Both the mean and range of FSRSs increased with time, on average by 2.5% every 5 years. Participants scored *M* = 27 (4.7 SD) out of 36 on total Hopkins Verbal Learning Test memory recall and *M* = 9 (2.7 SD) out of 12 on delayed memory recall (Table 3).

**Table 1:** Mean follow-up time between study waves and participant age at each wave

	1991–93	1997–99	2003–04	2007–09	2012–13	MRI scan: 2012–16
Time to scan (years): Mean (SD), Range	22 (1.4) 18–25	16 (1.4) 13–19	10 (1.4) 7–13	5 (1.4) 3–8	1 (1.3) 0–4	NA
Age (years): Mean (SD), Range	47.9 (5.2) 39.7–62.7	53.6 (5.2) 45.3–67.5	59.1 (5.2) 50.5–72.6	64.0 (5.2) 55.6–77.8	68.1 (5.2) 59.8–81.8	69.8 (5.2) 60.3–84.6
Age (years): Median	46.7	52.4	58.0	62.9	66.9	68.8

NA = not applicable; SD = standard deviation.

**Table 2:** Sociodemographic characteristics

	*N*	Verio and Prisma	*N*	Verio sample	*N*	Prisma sample	*N*	VBM analysis	*N*	TBSS analysis
Age (years): Mean (SD), Range	775	69.8 (5.2), 60.3–84.6	552	69.5 (5.3), 60.3–83.0	223	70.6 (4.8), 63.2–84.6	566	69.9 (5.2), 60.3–84.6	548	69.9 (5.2), 60.3–84.6
Sex: *N* (%), male	775	625 (80.6)	552	444 (80.4)	223	181 (81.2)	566	450 (79.5)	548	435 (79.4)
Ethnicity:										
White *N* (%)	775	733 (94.6)	552	516 (93.5)	223	217 (97.3)	566	534 (94.3)	548	517 (94.3)
Occupation: *N* (%)										
Administrative (highest)	775	320 (42.4)	552	234 (42.4)	223	86 (38.6)	566	230 (40.6)	548	220 (40.1)
Professional/executive	775	399 (52.9)	552	273 (49.5)	223	126 (56.5)	566	292 (51.6)	548	286 (52.2)
Clerical/support (lowest)	775	56 (7.4)	552	45 (8.2)	223	11 (4.9)	566	44 (7.7)	548	42 (7.6)
Education (years): Mean (SD), Range	775	14.05 (3.1), 6–23	552	14.00 (3.1), 6–23	223	14.20 (3.05), 6–22	566	13.97 (3.01), 6–23	548	14.01 (3.0), 6–23
Framingham stroke risk (%): Mean (SD), Range										
1991–94	709	3.32 (1.4), 1–13	502	3.32 (1.4), 1–13	207	3.29 (1.4), 1–13	566	3.27 (1.4), 1–13	548	3.26 (1.4), 1–13
1997–99	684	3.87 (2.3), 1–20	486	3.88 (2.3), 1–20	198	3.83 (2.2), 1–17	566	3.87 (2.3), 1–20	548	3.84 (2.3), 1–20
2002–04	718	5.35 (4.3), 1–52	508	5.50 (4.4), 1–52	210	5.00 (4.2), 1–37	566	5.29 (4.0), 1–29	548	5.18 (3.8), 1–29
2007–09	735	6.50 (4.9), 1–52	522	6.75 (5.2), 1–52	213	5.9 (3.92), 1–26	566	6.55 (5.0), 1–52	548	6.39 (4.5), 1–37
2012–13	743	8.50 (6.2), 1–64	524	8.76 (6.6), 1–52	219	7.89 (5.2), 1–29	566	8.59 (6.5), 1–64	548	8.50 (6.4), 1–64

SD = standard deviation; TBSS = tract-based spatial statistics.

**Table 3:** Memory performance and segmented brain values of the Verio and Prisma samples

	Verio and Prisma samples		Verio sample		Prisma sample	
	*N*	Mean (SD), range	*N*	Mean (SD), range	*N*	Mean (SD), range
HVLT-R (total recall)	775	27.4 (4.7), 10–36	552	27.5 (4.8), 10–36	223	27.2 (4.4), 15–35
HVLT-R (delayed recall)	775	9.2 (2.7), 0–12	552	9.2 (2.8), 0–12	223	9.1 (2.6), 0–12
Right hippocampus^a^ (mm^3^)	773	3700 (525), 1370–5412	550	3580 (493), 1370–5064	223	3998 (484), 2782–5412
Left hippocampus^a^ (mm^3^)	773	3651 (485), 1996–5305	550	3580 (468), 1996–5150	223	3826 (483), 2292–5305
Total intracranial volume^a^ (mm^3^)	773	1 589 563 (205 927), 860 447—2 242 966	550	1 656 169 (172 573), 860 447–2 242 966	223	1 425 286 (188 990), 914 417–1 926 628
Cerebrospinal fluid^b^ (mm^3^)	771	349 903 (64 992), 172 836–610 075	550	330 289 (56 983), 172 836–610 075	221	398 717 (57 667), 280 711–580 161
Grey matter^b^ (mm^3^)	771	557 938 (48 097), 415 276–707 896	550	552 412 (46 067), 415 276–707 896	221	571 690 (50 343), 452 639–703 112
White matter^b^ (mm^3^)	771	551 874 (59 735), 376 172–776 406	550	558 713 (60 122), 377 965–776 406	221	534 855 (55 325), 376 172–681 453
Intracranial volume^b^ (mm^3^)	771	1 459 718 (135 542), 1 023 552–1 927 776	550	1 441 418 (130 870), 1 023 552–197 776	221	1 505 262 (136 529), 1 118 270–1 798 853
Cortical atrophy (%)^b^ (mm^3^)	771	38.3 (2.0), 28.9–44.5	550	38.4 (2.0), 28.9–44.5	221	38.0 (2.0), 30.4–42.6
White matter hyperintensity volume^c^ (mm^3^)	770	0.46 (0.3), 0.08–2.47	549	0.42 (0.3), 0.08–2.5	221	0.55 (0.3), 0.27–2.34

a FreeSurfer.

b FAST.

c BIANCA.

HVLT-R = Hopkins Verbal Learning Test-Revise; SD = standard deviation.

### Voxel-based morphometry

**Figure 1:**
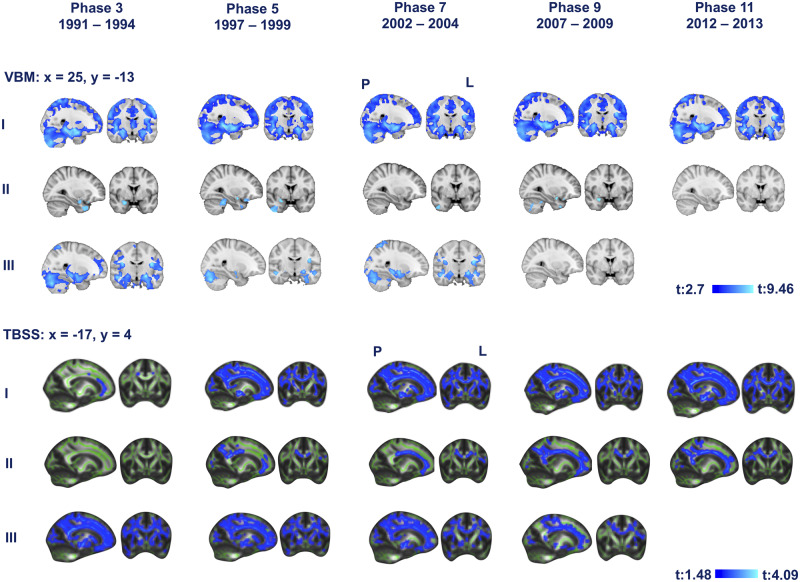
**The association of midlife Framingham stroke risk and lower grey matter density (top) and FA at older ages.** Rows I correspond to Model I (baseline model, uncorrected Framingham association with grey and white matter integrity). Rows II correspond to Model II (corrected model, Framingham stroke risk and grey and white matter integrity corrected for confounders). Rows III correspond to Model III (longitudinal model, analyses with significant results for associations between FSRS between 2012 and 2013, and voxelwise GM, and FA were repeated using scanner type and FSRS between 1991 and 1994, 1997 and 1999, 2002 and 2004 and 2007 and 2009 as a confounder). Blue represents regions significant at *P* < 0.05, threshold-free cluster enhancement, corrected for multiple comparisons. Coordinates are in MNI space. L = left; *M* = mean; P = posterior.

Model I: the FSRS in each of the five waves, even as early as 20 years preceding the MRI scan, was associated with widespread lower GM density at follow-up, across the cortex bilaterally, including higher cortical areas, the operculum and insular cortex, occipital cortex and medial temporal lobes (MTLs) (Fig. 1). Overall, FSRS measured in the earlier waves (at younger mean age) was associated with lower GM in as large a number of voxels, as risk measured at mean age 68 (5.2 SD) years. Peak cluster locations were in the right MTL and the amygdala [1991–94: *t* (563) = 3.27, *P *<* *0.001; 2002–04: *t* (563) = 3.24, *P *<* *0.001; 2007–09: *t* (563) = 3.38, *P *<* *0.001], right caudate [1997–99: *t* (563) = 3.36, *P* < 0.001] and left central opercular cortex [2012–13: *t* (563) = 3.29, *P* < 0.001, see Supplementary Table 2].

Model II: after adjusting for the confounders, FSRS measured at mean ages 47.9, 53.6, 59.1 and 64.0 (5.2 SD) years remained significantly associated with lower GM values, although the number of significant voxels was markedly reduced (Fig. 1, Supplementary Table 2). Results were localized in the cerebellar hemisphere, right MTL, middle temporal gyrus and frontal orbital cortex. A higher number of statistically significant voxels than in other waves were found with stroke risk measured on average 6 years before the scan. Maximum *t*-values were located in the temporal pole [1991–94: *t* (557) = 5.07, *P* = 0.008; 1997–99: *t* (557) = 6.05, *P* = 0.011; 2002–04: *t* (557) = 5.47, *P* = 0.018; 2007–09: *t* (557) = 5.13, *P* = 0.011, see Supplementary Table 2].

Model III: after removing the contribution of FSRS at mean ages 47.9, 53.6 and 59.1 (5.2 SD) years, FSRS between 2012 and 2013 [at mean age 68.1 (5.2 SD) years] predicted lower GM density in the MTLs. New FSRS risk acquired between the youngest and oldest study waves was associated with most widespread lower GM [*t* (563) = 2.96, *P* < 0.001]. After removing the contribution of FSRS during the penultimate study wave at mean age 64 (5.2 SD) years, FSRS between 2012 and 2013 [at mean age 68.1 (5.2 SD) years] did ‘not’ predict lower GM density.

### Tract-based spatial statistics

Model I: the FSRS at each of the five waves, at mean ages 47.9, 53.6, 59.1, 64.0 and 68.1 (5.2 SD) years and as early as 20 years preceding the brain scan, was associated with widespread lower FA at follow-up. FSRS measured closest to the time of scan, at mean age 68.1 (5.3 SD) years, was associated with the most widespread effect, including anterior thalamic radiation, cingulum, anterior and superior corona radiata, corpus callosum, corticospinal tract, external capsule, forceps major, forceps minor, internal capsule and superior longitudinal fasciculus. Peak cluster location was in the left corona radiata and forceps minor [*t* (547) = 2.5, *P* < 0.001]. The association was similar for higher MD, peak cluster location in left corona radiata [*t* (547) = 3.34, *P *<* *0.001].

Model II: after controlling the association for the confounders, FSRS measured at four time points between 1997 and 2013 [at mean ages 53.6, 59.1, 64.0 and 68.1 (5.2 SD) years] remained significantly associated with lower FA and higher MD values, although the number of significant voxels was markedly reduced. Associations were localized to the corpus callosum, longitudinal fasciculus, cingulate gyrus and anterior thalamic radiation (Fig. 1, Supplementary Table 3). FSRS closest to the time of scan (2012–13) remained the best predictor of lower FA and higher MD. Maximum *t*-values were located in the left cingulum [FA: *t* (541) = 1.8, *P* = 0.015, MD: *t* (541) = 2.94, *P* = 0.045].

Model III: after removing the contribution of FSRS at mean ages 47.9, 53.6, 59.1, 64.0 and 68.1 (5.2 SD) years, FSRS between 2012 and 2013 [at mean age 68.1 (5.2 SD) years] predicted lower FA and higher MD in the cingulum, anterior corona radiata, corpus callosum, longitudinal fasciculus and posterior thalamic radiation. The most widespread association between new FSRS risk and lower WM integrity was acquired between the youngest and oldest ages [FA: *t* (546) = 2.42, *P* < 0.001, and MD: *t* (546) = 3.28, *P* < 0.001].

**Figure 2:**
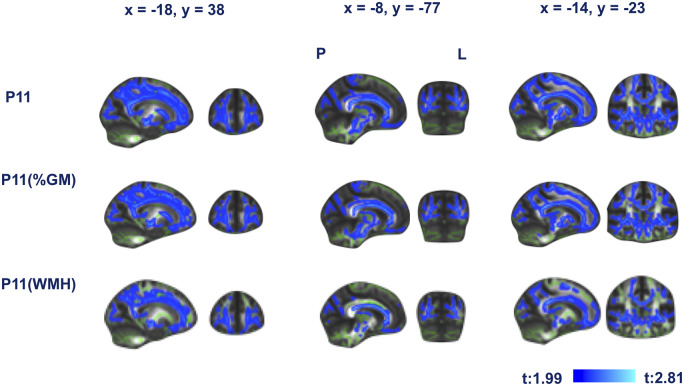
**Framingham stroke-risk predicted changes in white matter microstructure (FA) are primary to white matter lesions and secondary to Wallerian degeneration.** First row shows lower FA associated with Framingham stroke risk. Second row shows first row controlled for percentage grey matter (an estimate of Wallerian degeneration). Third row shows first row controlled for white matter hyperintensity volume. Blue represents regions significant at *P* < 0.05, threshold-free cluster enhancement, corrected for multiple comparisons. Coordinates are in MNI space. L = left; P = posterior.

Model IV: the association of FSRS measured at mean age 68.1 (5.2 SD) years and lower FA remained significant after controlling for GM [*t* (546) = 2.1, *P* < 0.001] and after controlling for WMH volume both as percentages of whole-brain volume [*t* (546) = 2.03, *P* < 0.001] (Fig. 2).

### Scanner differences

Voxelwise analyses were also performed separately on data acquired with the Verio and Prisma scanners, yielding results in the same locations, as previously found in a sample of Verio participants (Zsoldos, 2017). Sample characteristics, segmented GM and WMHs of both samples are presented in Tables 2 and 3.

### Structural equation modelling

**Figure 3:**
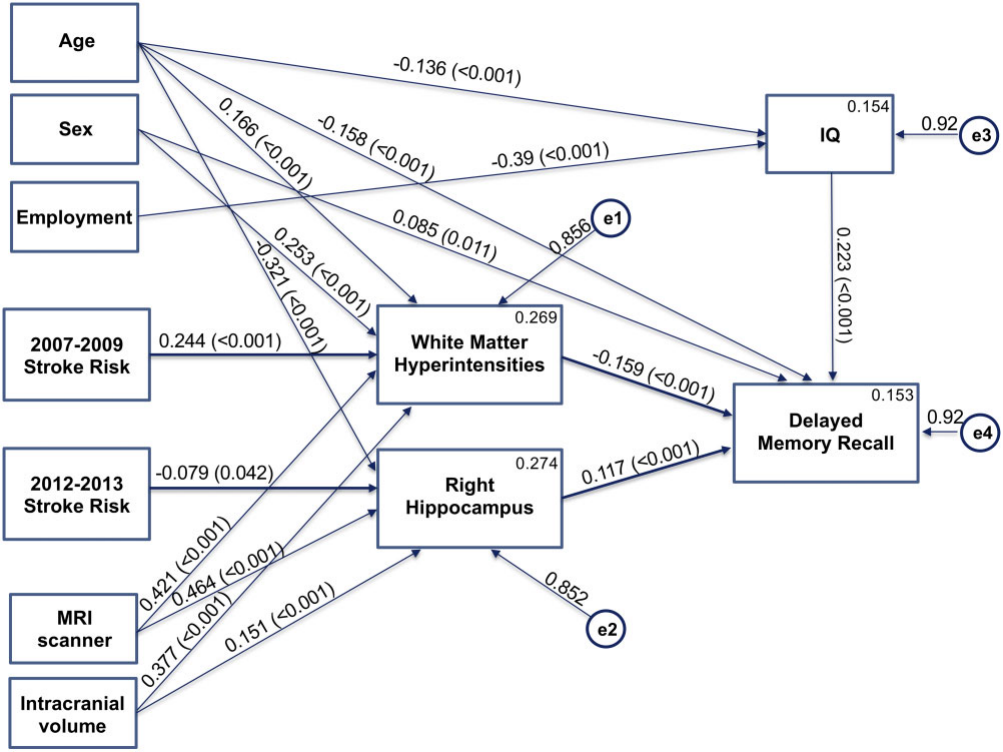
**Structural equation modelling results.** Framingham stroke risk in later waves was best associated with white matter hyperintensity and hippocampal volume, which, in turn, was associated with memory performance in older life. Covariances are not shown.

The structural equation modelling used 18 variables. Log-transformed FSRS between 1991 and 1994, 1997 and 1999, 2002 and 2004, 2007 and 2009 and 2012 and 2013, employment grade, age at scan, sex, scanner type and FreeSurfer-estimated total intracranial volume were entered as observed, exogenous variables (independent variables). WMH volume, right hippocampus, full-scale IQ (FSIQ; estimated from the Test of Premorbid Functioning) and delayed memory recall were entered as observed, endogenous variables (dependent variables). Each endogenous variable had an error term (unobserved, exogenous variable) with mean set to 0, and variance to 1. Covariances between exogenous variables and direct effects between each exogenous and endogenous variables were also modelled. Non-significant direct effects were removed until a suitable model fit of CMIN/DF <3 was achieved. The final model was significant with *χ*^2^ = 74.062 (df = 36, *P* < 0.001, *χ*^2^/df = 2.06). Higher 2007–09 FSRS predicted higher WMH volume, and higher 2012–13 FSRS predicted lower right hippocampal volume—both of which in turn predicted poorer delayed memory recall. Other significant direct effects were as follows: (i) higher age with higher WMH, lower hippocampal volume, FSIQ and delayed recall, (ii) women had higher WMH and delayed memory recall, (iii) participants scanned with the 3-T Prisma had higher WMH and right hippocampal volume, (iv) higher intracranial volume (ICV) with higher WMH and right hippocampal volume, (v) higher employment grade with higher FSIQ and (vi) higher FSIQ with higher delayed recall (Fig. 3, Supplementary Tables 4 and 5).

As we were interested in direct and indirect (via anatomical measures) effects of FSRS on delayed memory recall, we also evaluated a number of models including the direct effects of FSRS on delayed memory. Presumably, due to the collinearity of the FSRS measured at different time points (Pearson’s correlation between FSRS between 2007 and 2009 and between 2012 and 2013 = 0.826, *P* < 0.001) and the consequent different signs of effects, the sum of absolute direct and indirect effects did not add up to the absolute total effect, making interpretations difficult (Hayes, 2018). After removing the values for times not significantly associated with anatomical measures (leaving 2012–13 for hippocampal size and 2007–09 for WMH volumes), direct and indirect effects added up to total effects. In particular, the model retaining 2012–13 FSRS only achieved a *χ*^2^ = 74.100 (df = 18, *P* < 0.001, *χ*^2^/df = 4.12) and estimated direct effects of FSRS on Hopkins Verbal Learning Test as *β* = −0.226 and indirect effects via hippocampal size as *β* = −0.036, i.e. as 86% and 14%, respectively. The model retaining 2007–09 FSRS values only achieved a *χ*^2^ = 60.804 (df = 18, *P* < 0.001, *χ*^2^/df = 3.38) and estimated direct effects of FSRS on Hopkins Verbal Learning Test as *β* = −0.002 and indirect effects via volume of WMHs as *β* = −0.180, i.e. as 2% and 98%, respectively.

## Discussion

In this large group of community-dwelling older adults, unadjusted higher Framingham stroke risk measured across a 20-year period prior to the MRI scan was associated with lower whole-brain GM density and WM integrity. Higher stroke risk in younger ages, as early as 20 years before the MRI scan predicted lower GM density across the cortex and sub-cortical areas, and FA in corpus callosum. After removing the effect of confounding variables, including linear and squared age effects, higher stroke risk during earlier, but not at the most recent study wave, was associated with lower GM in the MTL. In addition, residual stroke risk over the 20-year period was associated with lower FA in widespread tracts, and lower GM density, confined mainly to sub-neocortical structures. Higher stroke risk at mean ages of 64 and 68.1 (5.2 SD) years statistically predicted lower Hopkins Verbal Learning Test-measured delayed memory performance. WMH volumes largely (90%) mediated this effect, while hippocampal volume did so only marginally.

### Grey matter

Our findings suggest that the unique effects of 10-year stroke risk impact GM density in (right) medial temporal structures up to old age. This means that FSRS does not only predict established clinical stroke but also ‘subclinical’ GM atrophy, particularly in MTL structures that may be of relevance in the development of cognitive impairment (Bastos-Leite *et al.*, 2007), depressive disorder (Santos *et al.*, 2018), Alzheimer’s disease (Burton *et al.*, 2009; den Heijer *et al.*, 2010) and dementia of vascular type (Laakso *et al.*, 1996). The effects of FSRS are independent of chronological age, which by itself predicts GM atrophy (Fotenos *et al.*, 2008) and the related cognitive decline in the general population (Jack *et al.*, 2005; Craik and Bialystok, 2006) and in the Whitehall II cohort (Singh-Manoux *et al.*, 2012). We added a quadratic age term because the effects of FSRS were still present after removing the effects of chronological age. This did not remove all the effects of FSRS, so FSRS makes a unique contribution to lower GM density, presumably based on vascular pathology beyond a simple function of time (Uiterwijk *et al.*, 2018). In a recent study of a subset (*N* = 116) of the Whitehall II imaging sub-study, we found that cardiovascular risk in midlife was significantly associated with lower GM perfusion at older ages, whereas this association was not significant for cardiovascular risk in later life, which lends further support to these results (Suri *et al.*, 2019). FSRS is a composite score and is incremental with age, which makes it difficult to estimate when it starts to affect GM, and when its effects become evident. FSRS at mean age 68.1 (5.2 SD) years, after removing the contribution of FSRS at mean ages of 47.9, 53.6 and 59.1 (5.2 SD) years, predicted lower GM density in the MTLs. This may mean that, while cardio-metabolic risk in the late 40s already predicts lower GM density at the age of 70 years in ‘neocortical areas’ located in middle cerebral artery vascular territories, which is the largest branch of the internal carotid and most often occluded by embolism, ‘sub-neocortical areas’ remain sensitive to residual risk over the following 20-year period. This is consistent with our knowledge that hippocampi remain plastic in adulthood and can regenerate (Spalding *et al.*, 2013; Bergmann *et al.*, 2015).

### White matter

FSRS in most data waves was associated with widespread lower microstructural WM integrity. FSRS measured 20 years prior to scan and, after the removal of confounders, was only associated with changes in the body of corpus callosum, superior corona radiata and their associated tracts in the right hemisphere. Change in stroke risk over a 20-year period was associated with lower FA in all major tracts. The findings that the associations were significantly reduced after removing confounding effects suggest that a large amount of variance is shared between WM microstructure and demographic factors. WM changes that are visible in fluid-attenuated inversion recovery images are generally interpreted clinically as microvascular changes (Debette and Markus, 2010). FSRS was predictive of WMHs in the present study as in previous ones (Jeerakathil *et al.*, 2004; Uiterwijk *et al.*, 2018). In our study, the effect of FSRS on WM microstructure was primarily mediated by widespread WM changes that are visible in fluid-attenuated inversion recovery images but was to some extent also secondary to Wallerian degeneration, suggesting that GM changes might not be of primary importance in generating the loss of WM integrity. WMHs affect cognitive abilities in healthy individuals (Debette *et al.*, 2010) and may also be related to the late-onset depression and the maintenance of impaired cognitive function in late-life depression (Kohler *et al.*, 2010).

### Cognition

Stroke and dementia have common risk factors, and there is strong evidence for the link between vascular risk factors and cognitive impairment (Elkins *et al.*, 2004; van Oijen *et al.*, 2007). Contrary to expectations (Pase *et al.*, 2018), FSRS in later but not in earlier waves was directly associated with higher WMH volume and lower hippocampal volume, which, in turn, was associated with worse memory performance in later life. Moreover, the effects of FSRS on verbal memory mediated by hippocampal size were relatively small, while the effect mediated via WMHs was accounting for almost all of the effect of FSRS on memory. Previous studies have found that vascular risk was a useful predictor of higher WMH burden and lower overall cognitive performance (Uiterwijk *et al.*, 2018) and have concluded that lowering vascular risk in midlife can potentially prevent dementias (Gorelick *et al.*, 2011; Hachinski and World Stroke Organization, 2015; Pase *et al.*, 2017). Furthermore, it is possible that Alzheimer’s biomarkers and cardiovascular risk predict cognitive performance through independent pathways (Buckner, 2004; Hohman *et al.*, 2015). The hippocampus is highly sensitive to cardiovascular and age-related damage or impaired regeneration (Nagy *et al.*, 1996; Molendijk *et al.*, 2012). However, it is likely that the stroke-risk effects are not only vascular in nature. Our voxelwise results support this finding, as neither an index of Wallerian degeneration nor WMHs could fully explain the association between FSRS and WM microstructure.

Strengths of this study are the 22-year repeated prospective data on Framingham stroke risk, the availability of a large amount of MRI data and the advanced methods of imaging analysis. The main limitation is that longitudinal MRI data are not available in parallel with the repeated measures of FSRS. However, the prospective association between FSRS assessed at mean age 47.9 (5.2 SD), two decades prior to the scan, with imaging measures highlighting the potential for targeting modifiable risk factors in midlife.

Findings of this study show that the Framingham 10-year probability stroke-risk score may relevant for primary prevention not only for stroke risk itself but also for subclinical GM atrophy in younger ages, and as early as 20 years in advance, and subsequent memory changes in later life. Brain areas such as the MTL are often implicated in cognitive impairment and dementias; thus, preserving them into older age is important. In the future, we may be able to use imaging results to track and target the modification of risk factors throughout adulthood.

## Supplementary Material

fcaa026_Supplementary_Data

## Abbreviations

FA =: fractional anisotropy
FSRS =: Framingham stroke-risk score
GM =: grey matter
MD =: mean diffusivity
MRI =: magnetic resonance imaging
MTL =: medial temporal lobe
WM =: white matter
WMH =: white matter hyperintensity
