# Supplementary material for: Association of midlife stroke risk with structural brain integrity and memory performance at older ages: a longitudinal cohort study
Source: Brain Commun. 2020 Mar 7;2(1):fcaa026. doi: 10.1093/braincomms/fcaa026 (PMC7491431; doi:10.1093/braincomms/fcaa026)
Supplement: fcaa026_Supplementary_Data [file fcaa026_supplementary_data.pdf]

Association of mid-life stroke risk with structural brain integrity and memory performance at older ages: A longitudinal cohort study.

Enikő Zsoldos*[1,2,3], DPhil, Abda Mahmood[1], MSc, Nicola Filippini[1,2,3], DPhil, Sana Suri[1,3], DPhil, **Verena Heise, DPhil[1,6],** Ludovica Griffanti[2], PhD Clare E Mackay[1,3], PhD, Archana Singh-Manoux[4,5], PhD, Mika Kivimäki[4], FMedSci, Klaus P Ebmeier[1], MD

[1]Department of Psychiatry, University of Oxford, Warneford Hospital, Oxford, OX3 7JX, UK
[2]Wellcome Centre for Integrative Neuroimaging, Oxford Centre for Functional MRI of the Brain, Nuffield Department of Clinical Neurosciences, University of Oxford, John Radcliffe Hospital, Oxford, OX3 9DU, UK
[3]Wellcome Centre for Integrative Neuroimaging, Oxford Centre for Human Brain Activity, University of Oxford, Warneford Hospital, Oxford, OX3 7JX, UK
[4]Department of Epidemiology and Public Health, University College London, London, WC1E 7HB, UK
[5]Centre for Research in Epidemiology and Population Health, INSERM, U1018, France
[6]Nuffield Department of Population Health, University of Oxford, Big Data Institute, Oxford, OX3 7LF, UK

*Corresponding Author: Dr Enikő Zsoldos, Department of Psychiatry, University of Oxford, Warneford Hospital, Oxford, OX3 7JX, UK, e-mail: eniko.zsoldos@psych.ox.ac.uk

Participant inclusion/ exclusion

VBM analysis was based on a final available sample of N = 566, TBSS on N = 548, and SEM on N = 775. Participants with no (N = 25) or inadequate quality T_1-weighted scan (N = 1), missing (N = 12) or un-useable dMRI scan (N = 6), and missing Framingham stroke risk score at any of the five data waves (N = 196), were excluded from whole-brain analysis. Following careful inspection of all T_1-weighted, FLAIR and dMRI images by an old-age psychiatrist with experience in neuroimaging (KPE), scans were excluded from whole-brain analysis due to the following structural abnormalities: large cyst (N = 7), meningioma (N = 1), infarction or stroke (N = 4) obvious on a T_1-weighted and FLAIR image. Incidental findings were further dealt with according to the FMRIB Centre internal protocol and processed by the associated neuroradiologists and neurologists without the involvement of the research team. At the time of scan, 18 participants reported a suspected stroke or TIA event. Out of these, 11 had a missing FSRS score (by definition the score can only be computed to predict a first stroke) and were removed from whole-brain analysis. Four participants had sub-optimal quality T1 images that could not be segmented by FAST (total N = 771), two by FreeSurfer (N = 773), and five by BIANCA (N = 770). One participant did not have a FLAIR scan therefore the WMH volume was imputed by regression (regressing WMH on age, sex, ethnicity, scanner type, years of education, socioeconomic status and FSRS between 2012-2013, to derive the regression coefficients, which can be used to calculate the WMH volume).

MRI acquisition and analysis

Cortical atrophy: Brain images were segmented and the total grey matter (GM), white matter (WM) and cerebrospinal fluid (CSF) volumes were extracted using FAST and FreeSurfer. FAST provides a partial volume image for each class, where each voxel contains a value in the range 0-1 that represents the proportion of that class's tissue present in that voxel (Zhang *et al.*, 2001). Cortical atrophy was estimated by scaling the GM values for the total intracranial volume (GM + WM + CSF)

resulting in percentage total GM volume. Total intracranial volume (TIV) was estimated with FreeSurfer version 5.3.

Hippocampal volume: FreeSurfer is a set of tools for a fully automated structural imaging analysis and visualization of brain imaging data. The volume-based subcortical stream of version 5.3 was used to pre-process MRI volumes and label subcortical tissue classes using the DKT atlas (?h.aparc.DKTatlas40.annot) (Fischl *et al.*, 2002). Hippocampal volume was used in the current analysis.

Grey matter density: Voxelwise analysis of GM was performed using FSL-VBM (Douaud *et al.*, 2007) an optimised voxel-based morphometry (VBM) protocol (Good *et al.*, 2001). Normalized bias-corrected brain extracted images were grey matter segmented before being registered to the Montreal Neurological Institute (MNI) 152 standard space using FMRIB's non-linear registration tool (FNIRT (Andersson *et al.*, 2007)). The images were averaged and flipped along the x-axis to create a left-right symmetric, study-specific GM template. All native GM images were non-linearly registered to this study-specific template and modulated to correct for local expansion (or contraction) due to the non-linear component of the spatial transformation. The modulated GM images were then smoothed with an isotropic Gaussian kernel with a sigma of 3 mm. Finally, a study-specific GM mask was used to enable accurate localization of results in an ageing sample.

White matter microstructure. Diffusion tensor imaging (DTI) quantifies the directionality and rate of diffusion of water molecules within different tissues and allows inferences about the structural integrity of WM tracts. When movement is anisotropic, such as in healthy myelinated fibres, diffusion is restricted perpendicular to the longitudinal axis of the fibre. DTI images were corrected for susceptibility-induced distortions using two b=0 scans (b-value 0 s/mm^2), acquired with opposing phase-encoding directions using the FSL 'topup' tool (Andersson *et al.*, 2003). Eddy current-induced distortions and subject movement were corrected using the FSL 'eddy' tool (Andersson and Sotiropoulos, 2016). This employs a second order polynomial model and transforms each scan towards the Gaussian process predicted scan. It also identifies outlier slices (dropout) caused by

movement during diffusion encoding (Andersson *et al.*, 2016). Slices were classified as outliers and replaced if the signal was found to be more than 3 SD from the Gaussian Process predicted slice. The volume was removed if over 10 slices were identified as outliers within a volume. The scan was excluded from analysis if more than five volumes were removed. Voxelwise statistical analysis of fractional anisotropy (FA) and mean diffusivity (MD) was carried out using tract-based spatial statistics ((TBSS)(Smith *et al.*, 2006)). A tensor model was fitted to the pre-processed diffusion data using DTIFit part of FMRIB's Diffusion Toolbox (http://fsl.fmrib.oc.ac.uk/fsl/fdt) to create FA and MD maps. This fits a diffusion tensor model to the pre-processed diffusion data and then brain-extracts using BET (Smith, 2002). All participant's FA data were then aligned into a common space using FNIRT (Andersson *et al.*, 2007), which uses a b-spline representation of the registration warp field (Rueckert *et al.*, 1999). Next, the mean FA image was created and thinned to create a mean FA skeleton, which represents the centers of all tracts common to the group. Each participant's aligned FA data were then projected onto this skeleton. The same non-linear warps and skeleton projections calculated for FA were then applied to MD. The resulting skeletonized FA and MD data were fed into voxelwise cross-subject statistics.

White matter hyperintensity (WMH) **volume.** WMHs were automatically segmented on FLAIR images with FMRIB's Brain Intensity AbNormality Classification Algorithm (BIANCA; (Griffanti *et al.*, 2016)). BIANCA is a fully automated, supervised method for WMH detection, based on the k-nearest neighbour algorithm. It classifies the image's voxels based on their intensity and spatial features, where the intensity features are extracted from FLAIR, T_1 and fractional anisotropy (FA) images. BIANCA offers options for weighting the spatial information, local intensity averaging and the choice of the number and location of training points. The following additional options were used: local average intensity within a 3D kernel of size = 3 voxels, MNI coordinates as spatial features with a weighting factor of 2, and 2000 lesion points and 10000 non-lesion points (avoiding the lesion border) for each of the 24 manually segmented images used as training dataset. The output image represents the probability per voxel of being WMH. The total WMH volume was calculated from

voxels exceeding a probability of 0.9 of being WMH located within a white matter mask and then adjusted for the total intracranial volume to obtain a percentage WMH volume.

Framingham Stroke Risk score

Risk components were drawn from both questionnaires and clinical examinations at 5 waves between 1991 and 2013. Risk components were collected according to standard operating protocols (Kivimaki *et al.*, 2012; Kaffashian *et al.*, 2013) detailed below.

Biological measures and samples were collected according to standard operating protocols (Kivimaki *et al.*, 2012; Kaffashian *et al.*, 2013). Systolic blood pressure was measured twice in sitting position after five minutes rest with a Hawskley random-zero sphygmomanometer (phases 3 and 5; Lynjay Services Ltd, Worthing, UK) and OMROM HEM 907 (phase 7 onwards; Omron, Milton Keynes, UK). The average of two readings was used in analysis. Atrial fibrillation (AF) and left ventricular hypertrophy (LVH) were identified on a standard 12-lead electrocardiogram analysis programme combined with manual review and the Minnesota code classification system for electrocardiographic findings (AF arrhythmias: 8-3-1; LVH high amplitude R-waves: 3-1) (Prineas *et al.*, 1982). History of prior cardiovascular disease (CVD) was based on electrocardiogram and angiogram examinations at phases 1, 3 and 5 as well as from general practitioner or hospital records. Hypertensive medication use was self-reported. Venous blood was taken in the fasting state or at least five hours after a light, fat-free breakfast before undergoing a standard 2h oral glucose tolerance test. Glucose was measured in fluoride plasma by an electrochemical glucose oxidase method (Cooper, 1973) on YSI model 23A glucose analyser at Phase 3 (Alpert, 1976) and YSI model 2300 STAT PLUS analyser at Phase 5 onwards (Astles *et al.*, 1996) (YSI Corporation, Yellow Springs, OH, USA). Diabetes mellitus was defined by a fasting glucose level of ≥ 7.0 mml/L, a 2hr post-load glucose level of ≥ 11.1 mml/L, self-reported diabetes diagnosed by a doctor or use of diabetes medication (Expert Committee on the and Classification of Diabetes, 2003). Participants were categorised as current cigarette smokers or past/non-smokers.

Assessment of cognition and premorbid functioning

The Hopkins Verbal Learning Test- Revised (HVLT-R) provides a measure of verbal learning and memory ability (1999; 2005), where the participant is required to learn a list of twelve words over the course of three trials, and recall or recognise them at increasing time intervals. The sum of items recalled after a 30-minute period (Total Delayed Recall; HVLT-DR (Wechsler, 2011)) was used for analysis.

The Test of Premorbid Functioning (TOPF)(Wechsler, 2011) consists of a list of seventy written words, which must be read aloud and is marked according to pronunciation. It is used to estimate an individual's level of intellectual functioning (full scale intelligence quotient) before the onset of injury or illness.

Supplementary Table 1. Acquisition parameters of T1-weighted, FLAIR and dMRI images on Siemens 3T Verio and Prisma scanners. FLAIR, fluid attenuated inversion

**Sequence**	**MEMPR**		**FLAIR**		**dMRI**	
Scanner	Verio	Prisma	Verio	Prisma	Verio	Prisma
TR, *ms*	2530	1900	9000	9000	8900	8900
TE, *ms*	1.79 / 3.65 / 5.51 / 7.37	3.97	73	73	91.2	91
Flip angle	7°	8°	150°	150°	-----	-----
Voxel, mm^3	1 x 1 x 1	1 x 1 x 1	0.9 x 0.9 x 3	0.4 x 0.4 x 3	2 x 2 x 2	2 x 2 x 2
FoV read	256	192	220	220	192	192
FoV phase	100%	100%	100%	100%	100%	100%
Base resolution	256	192	256	256	96	96
Phase resolution	100%	100%	100%	100%	100%	100%
TI, *ms*	1380	904	2500	2500	-----	-----
Bandwith, *Hz/Px*	651	200	283	283	1680	1680
Orientation	Sagittal	Transversal	Transversal	Transversal	Transversal	Transversal
b-value, s/mm^2	-----	-----	-----	-----	1500	1500
Directions, *N*	-----	-----	-----	-----	60 + 5 b=0	60 + 5 b=0
Acquisition time	6'12"	5'31"	4'14"	4'14"	10'32"	10'41"

recovery; dMRI, diffusion MRI; TR, repetition time; TE, echo time; FoV, field of view; TI, inversion time, Acquisition T; acquisition time.

Supplementary Table 2**.** Significant clusters of voxelwise grey matter negatively associated with Framingham Stroke Risk score (Models I – III).

Model I: FSRS 10yr probability								
**Data wave**	**Cluster**	**Cluster size (Voxels)**	**Max t**	**p - value**	**MNI coordinates (x,y,z)**			**Structure associated with max effect**
**1991 - 1993**	1	105,677	3.27	< 0.001	30	0	-16	R amygdala
	2	30	2.7	0.045	22	-8	50	R superior frontal gyrus
	3	17	3.04	0.042	-16	48	-2	L frontal medial cortex, frontal pole
**1997 - 1999**	1	120,937	3.36	< 0.001	2	4	0	R caudate
**2002 - 2004**	1	99,166	3.24	< 0.001	30	2	-18	R amygdala
**2007 - 2009**	1	111,849	3.38	< 0.001	30	2	-18	R amygdala
**2012 - 2013**	1	95,058	3.29	< 0.001	-38	-16	22	L central opercular cortex
	2	4	2.7	0.049	-18	-6	54	L superior frontal gyrus

Model II: Model I + confounding variables								
**Data wave**	**Cluster**	**Cluster size (Voxels)**	**Max t**	**p - value**	**MNI coordinates (x,y,z)**			**Structure associated with max effect**
**1991 - 1993**	1	511	5.07	0.008	34	10	-36	Temporal pole
	2	128	8.1	0.02	30	0	-16	R amygdala
	3	11	3.71	0.046	34	22	-16	Frontal orbital cortex
**1997 - 1999**	1	1,075	6.05	0.011	36	4	-34	Temporal pole
	2	839	8.09	0.024	48	-28	-4	Middle temporal gyrus, superior temporal gyrus
	3	680	9.16	0.014	24	-28	-12	R hippocampus
	4	113	5.9	0.03	-16	-34	2	L thalamus
	5	34	9.49	0.041	30	0	-16	R amygdala
	6	24	5.5	0.044	20	-74	-6	Lingual gyrus, occipital fusiform gyrus
**2002 - 2004**	1	172	5.47	0.018	40	6	-34	Temporal pole
**2007 - 2009**	1	2,115	8.35	0.012	-30	-76	-36	Cerebellar hemisphere
	2	515	5.13	0.011	38	20	-20	Frontal orbital cortex, temporal pole
	3	254	6.59	0.022	16	-90	-32	Cerebellar hemisphere
	4	110	6.16	0.039	36	-46	-32	
	5	107	7.43	0.042	30	-68	-44	
	6	45	9.1	0.038	30	2	-18	R amygdala
	7	36	6.33	0.038	20	-82	-14	Occipital fusiform gyrus
	8	28	4.61	0.043	32	12	-42	Temporal pole
	9	6	3.67	0.048	16	-86	0	Lingual gyrus, occipital fusiform gyrus

Model III: FSRS at 2012 - 2013 + FSRS at previous waves								
**Data wave**	**Cluster**	**Cluster size (Voxels)**	**Max t**	**p - value**	**MNI coordinates (x,y,z)**			**Structure associated with max effect**
**1991 - 1993**	1	48,138	2.96	< 0.001	-38	-16	22	Central opercular cortex, parietal operculum cortex
	2	753	3.34	0.004	10	-64	66	Lateral occipital cortex superior div., precuneous cortex
	3	432	2.87	0.013	-14	-54	72	Superior parietal lobule, lateral occipital cortex
	4	153	2.72	0.035	-12	-24	68	Precentral gyrus
	5	46	2.76	0.042	6	-16	68	Precentral gyrus, juxatopsitional lobule
	6	42	3	0.038	-18	-68	64	Lateral occipital cortex superior div., superior parietal lobule
	7	10	3.54	0.047	10	-80	40	Cuneal cortex, precuneous cortex
**1997 - 1999**	1	11,108	2.84	0.001	-24	-76	-14	Occipital fusiform gyrus, lingual gyrus
	2	1,803	2.71	0.011	-24	-12	-10	L amygdala, hippocampus
	3	538	3.19	0.015	52	-12	-14	Middle temporal gyrus, superior temporal gyrus
	4	357	3.48	0.014	-38	-16	22	Central opercular cortex, parietal operculum cortex
	5	38	3.59	0.041	32	-24	12	Insular cortex
	6	17	3.6	0.043	54	-24	48	Postcentral gyrus, supramarginal gyrus
**2002 - 2004**	1	28,264	2.57	0.001	16	-6	-12	R amygdala
	2	88	3.35	0.03	-12	-38	30	Cingulate gyrus

Supplementary Table 3. Significant clusters of voxelwise tract-based spatial statistics (TBSS) negatively associated with Framingham Stroke Risk score (Models I – IV).

**Model I: FSRS 10yr probability**									
**TBSS measure**	**Data wave**	**Cluster**	**Cluster size (Voxels)**	**Max t**	**p - value**	**MNI coordinates (x,y,z)**			**Structure associated with max effect**
**Fractional Anisotropy**	**1991 - 1994**	1	1,153	2.74	0.026	18	28	21	R anterior corona radiata, Forceps minor
		2	623	2.78	0.036	-20	36	2	L anterior corona radiata, anterior thalamic radiation, forceps minor
		3	10	3.51	0.049	-17	4	36	L superior corona radiata
	**1997- 1999**	1	64,519	2.31	< 0.001	-18	38	8	L anterior corona radiata, forceps minor
	**2002 - 2004**	1	74,156	2.39	< 0.001	-18	38	8	L anterior corona radiata, forceps minor
		2	42	4.09	0.046	5	-55	-16	R inferior cerebellar penducle
	**2007 - 2009**	1	75,538	2.56	< 0.001	-29	-56	15	L posterior thalamic radiation, forceps major
	**2012 - 2013**	1	80,676	2.5	< 0.001	-18	38	9	L anterior corona radiata, forceps minor
**Mean Diffusivity**	**1991 - 1994**	1	18,691	2.43	0.006	-12	-26	-4	L corticospinal tract
	**1997- 1999**	1	73,085	2.76	< 0.001	-12	-26	-4	L corticospinal tract
	**2002 - 2004**	1	77,854	3.03	< 0.001	-21	10	31	L superior corona radiata
	**2007 - 2009**	1	81,523	3.38	< 0.001	23	-25	31	R posterior corona radiata, corticospinal tract
	**2012 - 2013**	1	82,590	3.34	< 0.001	-20	11	31	L superior corona radiata

Model II: Model I + confounding variables									
TBSS measure	Data wave	Cluster	Cluster size (Voxels)	Max t	p - value	MNI coordinates (x,y,z)			Structure associated with max effect
Fractional Anisotropy	1997 - 1999	1	8,950	1.94	0.017	-40	-45	20	L superior longitudinal fasciculus
		2	91	2.38	0.048	-21	46	11	Forceps minor, L anterior thalamic radiation
	2002 - 2004	1	6,411	1.95	0.016	-11	-25	27	Body of corpus callosum
		2	140	2.49	0.048	-24	-73	14	Forceps major, L inferior fronto-occipital fasciculus
		3	125	1.82	0.05	-39	-2	32	L superior longitudinal fasciculus
		4	23	1.73	0.05	-40	-5	39	
		5	18	2.61	0.05	36	34	-2	R interior fronto-occipital fasciculus
	2007 - 2009	1	14,331	1.93	0.008	-19	-44	43	L cingulate gyrus (cingulum)
		2	333	2.54	0.045	23	-41	40	R cingulate gyrus (cingulum)
	2012 - 2013	1	13,109	1.8	0.015	-18	-44	42	L cingulate gyrus (cingulum)
		2	2,682	1.98	0.023	-25	-71	15	Forceps major, L inferior fronto-occipital fasciculus
		3	1,091	1.95	0.038	35	-2	22	L superior longitudinal fasciculus
		4	79	1.78	0.049	30	11	35	R superior longitudinal fasciculus
		5	43	2.55	0.049	-18	-67	39	L inferior longitudinal fasciculus
		6	34	2	0.049	33	21	35	R anterior thalamic radiation
		7	29	1.78	0.05	32	14	45	R superior longitudinal fasciculus
		8	17	3.21	0.049	36	33	-3	R interior fronto-occipital fasciculus
		9	15	3.42	0.049	50	2	18	R superior longitudinal fasciculus
		10	9	1.65	0.05	38	23	32	R interior fronto-occipital fasciculus
		11	6	3.05	0.05	42	27	-5	
		12	1	1.48	0.05	37	26	30	
		13	1	1.53	0.05	33	23	33	R anterior thalamic radiation

**Mean Diffusivity**	**1997 - 1999**	1	408	2.94	0.045	-19	30	19	Forceps minor, L cingulum (cingulate gyrus)
		2	336	2.99	0.046	-26	21	36	L superior longitudinal fasciculus
		3	184	2.92	0.047	21	25	23	R anterior thalamic radiation
		4	74	2.94	0.049	15	32	13	Forceps minor, Genu of corpus callosum
		5	14	3.18	0.05	11	31	4	
		6	11	5.4	0.05	31	4	46	R superior longitudinal fasciculus
		7	1	3.38	0.05	10	30	-1	Forceps minor, Genu of corpus callosum
	**2002 - 2004**	1	17,029	2.03	0.023	34	40	25	R superior longitudinal fasciculus
		2	278	2.22	0.048	-34	-14	-7	L external capsule, inferior fronto-occipital fasciculus
		3	8	3.64	0.05	25	16	-12	R uncinate fasciculus
	**2007 - 2009**	1	35,815	2.17	0.002	23	-25	31	R corticospinal tract
	**2012 - 2013**	1	15,949	2.07	0.01	-37	-8	51	L superior longitudinal fasciculus
		2	15,221	2.06	0.01	26	13	22	R anterior corona radiata
		3	118	2.64	0.048	-14	-18	4	L anterior thalamic radiation
		4	97	2.77	0.049	-10	-9	0	
		5	3	3.01	0.05	-5	-6	-3	

Model III: FSRS at 2012 - 2013 + FSRS at previous waves									
**TBSS measure**	**Previous data wave**	**Cluster**	**Cluster size (Voxels)**	**Max t**	**p - value**	**MNI coordinates (x,y,z)**			**Structure associated with max effect**
**Fractional Anisotropy**	**1991 - 1994**	1	88,036	2.42	< 0.001	-18	38	9	L anterior corona radiata, forceps minor
	**1997 - 1999**	1	67,644	1.94	< 0.001	28	-69	17	Forceps major
	**2002 - 2004**	1	53,287	1.87	0.001	-18	38	9	L anterior corona radiata, forceps minor
	**2007 - 2009**	1	16,517	1.74	0.015	21	-47	57	R superior longitudinal fasciculus, anterior thalamic radiation
		2	3,515	1.65	0.042	-5	-43	-24	L anterior thalamic radiation
		3	960	1.62	0.046	12	-53	22	R cingulum (cingulate gyrus, hippocampus)
		4	940	2.06	0.036	28	-69	17	Forceps major
		5	791	1.75	0.045	-50	-47	24	L superior longitudinal fasciculus
		6	330	2.18	0.047	43	-60	38	R superior longitudinal fasciculus
		7	140	1.81	0.049	12	56	20	Forceps minor
		8	75	2.06	0.049	-11	51	-14	
		9	68	1.83	0.049	12	47	34	
		10	56	2.51	0.049	36	-56	5	R posterior thalamic radiation, inferior fronto-occipital fasciculus
		11	34	1.91	0.049	22	-60	49	R superior longitudinal fasciculus
		12	30	2.08	0.049	-11	60	11	Forceps minor
		13	21	1.83	0.05	-16	54	6	
**Mean Diffusivity**	**1991 - 1994**	1	89,121	3.28	< 0.001	27	13	24	R anterior corona radiata, superior longitudinal fasciculus
	**1997 - 1999**	1	73,636	2.54	< 0.001	27	13	24	R anterior corona radiata, superior longitudinal fasciculus
	**2002 - 2004**	1	66,723	2.29	0.001	26	13	22	R anterior corona radiata
	**2007 - 2009**	1	37,077	1.92	0.009	-37	-8	51	L superior longitudinal fasciculus

Model IV: FSRS at 2012 - 2013 + percentage total GM or WMH volume									
**TBSS measure**		**Cluster**	**Cluster size (Voxels)**	**Max t**	**p - value**	**MNI coordinates (x,y,z)**			**Structure associated with max effect**
**Fractional Anisotropy**	**2012 - 2013**	1	80,676	2.5	< 0.001	-18	38	9	L anterior corona radiata, forceps minor
	**%GM**	1	74,429	2.1	< 0.001	-18	38	9	L anterior corona radiata, Forceps minor
	**WMH**	1	49,986	2.03	< 0.001	-18	38	9	L anterior corona radiata, Forceps minor
		2	432	2.04	0.039	-8	-77	21	Forceps major
		3	23	2.81	0.049	-14	-23	1	L corticospinal tract
		4	17	2.23	0.05	-9	35	13	L forceps minor, cingulum (cingulate gyrus)
		5	10	1.99	0.05	-6	-21	-28	L corticospinal tract
		6	8	2.34	0.05	-4	-21	-22	L anterior thalamic radiation, corticospinal tract
		7	2	2.79	0.05	-7	17	27	L cingulum (cingulate gyrus)
**Mean Diffusivity**	**2012 - 2013**	1	82,590	3.34	< 0.001	-20	11	31	L superior corona radiata
	**%GM**	1	79,240	2.92	< 0.001	-20	11	31	L superior corona radiata
	**WMH**	1	66,163	2.31	< 0.001	-20	11	31	L superior corona radiata

Supplementary Table 4**.** AMOS Structural Equation Modelling Regression Weights

			**Estimate**	**S.E.**	**C.R.**	**P-value**
WHITE MATTER HYPERINTENSITY VOLUME (BIANCA)	<---	FRAMINGHAM 10YR RISK OF STROKE 2007-2009 (LN)	1833.414	359.747	5.096	< 0.001
WHITE MATTER HYPERINTENSITY VOLUME (BIANCA)	<---	AGE AT SCAN	137.136	35.933	3.816	< 0.001
WHITE MATTER HYPERINTENSITY VOLUME (BIANCA)	<---	SEX	2754.711	468.884	5.875	< 0.001
WHITE MATTER HYPERINTENSITY VOLUME (BIANCA)	<---	SCANNER	4000.784	391.312	10.224	< 0.001
WHITE MATTER HYPERINTENSITY VOLUME (BIANCA)	<---	ESTIMATED TOTAL INTRACRANIAL VOLUME (FREESURFER)	0.008	0.001	8.438	< 0.001
WHITE MATTER HYPERINTENSITY VOLUME (BIANCA)	<---	E1	3675.894	101.887	36.078	< 0.001
RIGHT HIPPOCAMPUS VOLUME (FREESURFER)	<---	FRAMINGHAM 10YR RISK OF STROKE 2012-2013 (LN)	-70.698	34.838	-2.029	0.042
RIGHT HIPPOCAMPUS VOLUME (FREESURFER)	<---	AGE AT SCAN	-32.415	3.861	-8.395	< 0.001
RIGHT HIPPOCAMPUS VOLUME (FREESURFER)	<---	SCANNER	538.415	41.662	12.923	< 0.001
RIGHT HIPPOCAMPUS VOLUME (FREESURFER)	<---	ESTIMATED TOTAL INTRACRANIAL VOLUME (FREESURFER)	0.000	0.000	4.15	< 0.001
RIGHT HIPPOCAMPUS VOLUME (FREESURFER)	<---	E2	446.798	11.372	39.289	< 0.001
FSIQ (ESTIMATED FROM TOPF)	<---	AGE AT SCAN	-0.223	0.055	-4.077	< 0.001
FSIQ (ESTIMATED FROM TOPF)	<---	LUMPED SES LEVEL - 3 GROUPS (S1-D)	-5.456	0.468	-11.656	< 0.001
FSIQ (ESTIMATED FROM TOPF)	<---	E4	7.805	0.198	39.345	< 0.001
HVLT DELAYED RECALL	<---	AGE AT SCAN	-0.083	0.02	-4.151	< 0.001
HVLT DELAYED RECALL	<---	SEX	0.586	0.23	2.544	0.011
HVLT DELAYED RECALL	<---	FSIQ (ESTIMATED FROM TOPF)	0.071	0.011	6.65	< 0.001
HVLT DELAYED RECALL	<---	WHITE MATTER HYPERINTENSITY VOLUME (BIANCA)	0.000	0.000	-4.177	< 0.001
HVLT DELAYED RECALL	<---	RIGHT HIPPOCAMPUS VOLUME (FREESURFER)	0.001	0.000	3.319	< 0.001
HVLT DELAYED RECALL	<---	E3	2.505	0.064	39.193	< 0.001

Supplementary Table 5. AMOS Structural Equation Modelling Covariances

			Estimate	S.E.	C.R.	P-value
AGE AT SCAN	<-->	SCANNER	0.247	0.062	3.957	< 0.001
SEX	<-->	ESTIMATED TOTAL INTRACRANIAL VOLUME (FREESURFER)	-34070.143	2795.433	-12.188	< 0.001
LUMPED SES LEVEL - 3 GROUPS (S1-D)	<-->	SEX	0.075	0.009	8.421	< 0.001
LUMPED SES LEVEL - 3 GROUPS (S1-D)	<-->	AGE AT SCAN	-0.492	0.107	-4.588	< 0.001
LUMPED SES LEVEL - 3 GROUPS (S1-D)	<-->	ESTIMATED TOTAL INTRACRANIAL VOLUME (FREESURFER)	-29846.797	3966.454	-7.525	< 0.001
FRAMINGHAM 10YR RISK OF STROKE 1991-1994 (LN)	<-->	FRAMINGHAM 10YR RISK OF STROKE 1997-1999 (LN)	0.176	0.01	17.288	< 0.001
FRAMINGHAM 10YR RISK OF STROKE 1991-1994 (LN)	<-->	FRAMINGHAM 10YR RISK OF STROKE 2002-2007 (LN)	0.168	0.011	15.824	< 0.001
FRAMINGHAM 10YR RISK OF STROKE 1991-1994 (LN)	<-->	FRAMINGHAM 10YR RISK OF STROKE 2007-2009 (LN)	0.155	0.01	15.622	< 0.001
FRAMINGHAM 10YR RISK OF STROKE 1991-1994 (LN)	<-->	FRAMINGHAM 10YR RISK OF STROKE 2012-2013 (LN)	0.137	0.01	14.008	< 0.001
FRAMINGHAM 10YR RISK OF STROKE 1991-1994 (LN)	<-->	LUMPED SES LEVEL - 3 GROUPS (S1-D)	-0.054	0.01	-5.413	< 0.001
FRAMINGHAM 10YR RISK OF STROKE 1991-1994 (LN)	<-->	SEX	-0.138	0.008	-16.982	< 0.001
FRAMINGHAM 10YR RISK OF STROKE 1991-1994 (LN)	<-->	AGE AT SCAN	0.405	0.057	7.079	< 0.001
FRAMINGHAM 10YR RISK OF STROKE 1991-1994 (LN)	<-->	ESTIMATED TOTAL INTRACRANIAL VOLUME (FREESURFER)	31321.946	3087.801	10.144	< 0.001
FRAMINGHAM 10YR RISK OF STROKE 1997-1999 (LN)	<-->	FRAMINGHAM 10YR RISK OF STROKE 2002-2007 (LN)	0.257	0.014	18.815	< 0.001
FRAMINGHAM 10YR RISK OF STROKE 1997-1999 (LN)	<-->	FRAMINGHAM 10YR RISK OF STROKE 2007-2009 (LN)	0.223	0.012	17.964	< 0.001
FRAMINGHAM 10YR RISK OF STROKE 1997-1999 (LN)	<-->	FRAMINGHAM 10YR RISK OF STROKE 2012-2013 (LN)	0.214	0.013	16.99	< 0.001
FRAMINGHAM 10YR RISK OF STROKE 1997-1999 (LN)	<-->	LUMPED SES LEVEL - 3 GROUPS (S1-D)	-0.067	0.012	-5.804	< 0.001
FRAMINGHAM 10YR RISK OF STROKE 1997-1999 (LN)	<-->	SEX	-0.121	0.008	-14.925	< 0.001
FRAMINGHAM 10YR RISK OF STROKE 1997-1999 (LN)	<-->	AGE AT SCAN	1.152	0.09	12.747	< 0.001
FRAMINGHAM 10YR RISK OF STROKE 1997-1999 (LN)	<-->	ESTIMATED TOTAL INTRACRANIAL VOLUME (FREESURFER)	29909.999	3289.042	9.094	< 0.001
FRAMINGHAM 10YR RISK OF STROKE 2002-2007 (LN)	<-->	FRAMINGHAM 10YR RISK OF STROKE 2007-2009 (LN)	0.281	0.015	18.783	< 0.001
FRAMINGHAM 10YR RISK OF STROKE 2002-2007 (LN)	<-->	FRAMINGHAM 10YR RISK OF STROKE 2012-2013 (LN)	0.272	0.015	17.892	< 0.001
FRAMINGHAM 10YR RISK OF STROKE 2002-2007 (LN)	<-->	LUMPED SES LEVEL - 3 GROUPS (S1-D)	-0.064	0.013	-4.922	< 0.001
FRAMINGHAM 10YR RISK OF STROKE 2002-2007 (LN)	<-->	SEX	-0.101	0.008	-12.572	< 0.001
FRAMINGHAM 10YR RISK OF STROKE 2002-2007 (LN)	<-->	AGE AT SCAN	1.687	0.119	14.195	< 0.001
FRAMINGHAM 10YR RISK OF STROKE 2002-2007 (LN)	<-->	ESTIMATED TOTAL INTRACRANIAL VOLUME (FREESURFER)	25128.077	3561.925	7.055	< 0.001
FRAMINGHAM 10YR RISK OF STROKE 2002-2007 (LN)	<-->	SCANNER	-0.017	0.006	-3.028	0.002

FRAMINGHAM 10YR RISK OF STROKE 2007-2009 (LN)	<-->	FRAMINGHAM 10YR RISK OF STROKE 2012-2013 (LN)	0.276	0.015	18.614	< 0.001
FRAMINGHAM 10YR RISK OF STROKE 2007-2009 (LN)	<-->	LUMPED SES LEVEL - 3 GROUPS (S1-D)	-0.064	0.012	-5.197	< 0.001
FRAMINGHAM 10YR RISK OF STROKE 2007-2009 (LN)	<-->	SEX	-0.094	0.008	-12.495	< 0.001
FRAMINGHAM 10YR RISK OF STROKE 2007-2009 (LN)	<-->	AGE AT SCAN	1.654	0.114	14.52	< 0.001
FRAMINGHAM 10YR RISK OF STROKE 2007-2009 (LN)	<-->	ESTIMATED TOTAL INTRACRANIAL VOLUME (FREESURFER)	28927.775	3427.506	8.44	< 0.001
FRAMINGHAM 10YR RISK OF STROKE 2007-2009 (LN)	<-->	SCANNER	-0.018	0.006	-2.941	0.003
FRAMINGHAM 10YR RISK OF STROKE 2012-2013 (LN)	<-->	AGE AT SCAN	1.744	0.122	14.317	< 0.001
FRAMINGHAM 10YR RISK OF STROKE 2012-2013 (LN)	<-->	ESTIMATED TOTAL INTRACRANIAL VOLUME (FREESURFER)	22800.543	3468.923	6.573	< 0.001
FRAMINGHAM 10YR RISK OF STROKE 2012-2013 (LN)	<-->	SEX	-0.07	0.007	-9.682	< 0.001
FRAMINGHAM 10YR RISK OF STROKE 2012-2013 (LN)	<-->	LUMPED SES LEVEL - 3 GROUPS (S1-D)	-0.055	0.013	-4.33	< 0.001
FRAMINGHAM 10YR RISK OF STROKE 2012-2013 (LN)	<-->	SCANNER	-0.018	0.007	-2.603	0.009
ESTIMATED TOTAL INTRACRANIAL VOLUME (FREESURFER)	<-->	SCANNER	-47448.67	3441.705	-13.786	< 0.001

References

Construct and concurrent validity of the Hopkins Verbal Learning Test - Revised. Clin Neuropsychol 1999; 13(3): 348-58.

Test-retest reliability of component process variables within the Hopkins Verbal Learning Test-Revised. Assessment 2005; 12(1): 96-100.

Alpert L. Instrument series: model 23A Glucose Analyzer. Lab World 1976; 27: 8-13.

Andersson J, Jenksinson M, Smith S. Non-linear registration, aka Spatial normalisation. FMRIB technical report TR07JA2 from www.fmrib.ox.ac.uk/analysis/techrep; 2007.

Andersson JL, Skare S, Ashburner J. How to correct susceptibility distortions in spin-echo echo-planar images: application to diffusion tensor imaging. Neuroimage 2003; 20(2): 870-88.

Andersson JLR, Graham MS, Zsoldos E, Sotiropoulos SN. Incorporating outlier detection and replacement into a non-parametric framework for movement and distortion correction of diffusion MR images. Neuroimage 2016; 141: 556-72.

Andersson JLR, Sotiropoulos SN. An integrated approach to correction for off-resonance effects and subject movement in diffusion MR imaging. Neuroimage 2016; 125: 1063-78.

Astles JR, Sedor FA, Toffaletti JG. Evaluation of the YSI 2300 glucose analyzer: algorithm-corrected results are accurate and specific. Clin Biochem 1996; 29(1): 27-31.

Cooper GR. Methods for determining the amount of glucose in blood. CRC Crit Rev Clin Lab Sci 1973; 4(2): 101-45.

Douaud G, Smith S, Jenkinson M, Behrens T, Johansen-Berg H, Vickers J*, et al.* Anatomically related grey and white matter abnormalities in adolescent-onset schizophrenia. Brain 2007; 130(Pt 9): 2375-86.

Expert Committee on the D, Classification of Diabetes M. Report of the expert committee on the diagnosis and classification of diabetes mellitus. Diabetes Care 2003; 26 Suppl 1: S5-20.

Fischl B, Salat DH, Busa E, Albert M, Dieterich M, Haselgrove C*, et al.* Whole brain segmentation: automated labeling of neuroanatomical structures in the human brain. Neuron 2002; 33(3): 341-55.

Good CD, Johnsrude IS, Ashburner J, Henson RN, Friston KJ, Frackowiak RS. A voxel-based morphometric study of ageing in 465 normal adult human brains. Neuroimage 2001; 14(1 Pt 1): 21-36.

Griffanti L, Zamboni G, Khan A, Li L, Bonifacio G, Sundaresan V*, et al.* BIANCA (Brain Intensity AbNormality Classification Algorithm): A new tool for automated segmentation of white matter hyperintensities. Neuroimage 2016; 141: 191-205.

Kaffashian S, Dugravot A, Brunner EJ, Sabia S, Ankri J, Kivimaki M*, et al.* Midlife stroke risk and cognitive decline: a 10-year follow-up of the Whitehall II cohort study. Alzheimers Dement 2013; 9(5): 572-9.

Kivimaki M, Shipley MJ, Allan CL, Sexton CE, Jokela M, Virtanen M*, et al.* Vascular risk status as a predictor of later-life depressive symptoms: a cohort study. Biol Psychiatry 2012; 72(4): 324-30.

Prineas RJ, Crow RS, Blackburn HW. The Minnesota Code Manual for Electrocardiographic Findings: Standards and Procedures for Measurement and Classification. Bristol, UK.: John Wright; 1982.

Rueckert D, Sonoda LI, Hayes C, Hill DL, Leach MO, Hawkes DJ. Nonrigid registration using free-form deformations: application to breast MR images. IEEE Trans Med Imaging 1999; 18(8): 712-21.

Smith SM. Fast robust automated brain extraction. Hum Brain Mapp 2002; 17(3): 143-55.

Smith SM, Jenkinson M, Johansen-Berg H, Rueckert D, Nichols TE, Mackay CE*, et al.* Tract-based spatial statistics: voxelwise analysis of multi-subject diffusion data. Neuroimage 2006; 31(4): 1487-505.

Wechsler D. Test of Premorbid Functioning. UK Version (TOPF UK). Bloomington, MN: Pearson Inc; 2011.

Zhang Y, Brady M, Smith S. Segmentation of brain MR images through a hidden Markov random field model and the expectation-maximization algorithm. IEEE Trans Med Imaging 2001; 20(1): 45-57.
